# Multiscale and Multimodal Approaches to Study Autophagy in Model Plants

**DOI:** 10.3390/cells7010005

**Published:** 2018-01-09

**Authors:** Jessica Marion, Romain Le Bars, Laetitia Besse, Henri Batoko, Béatrice Satiat-Jeunemaitre

**Affiliations:** 1 Institute of Integrative Biology of the Cell (I2BC), CEA, CNRS, Université Paris-Sud, Université Paris-Saclay, 91 198 Gif-sur-Yvette, France; jessica.marion@i2bc.paris-saclay.fr (J.M.); romain.lebars@i2bc.paris-saclay.fr (R.L.B.); laetitia.besse@i2bc.paris-saclay.fr (L.B.); bsj@i2bc.paris-saclay.fr (B.S.-J.); 2 Institute of Life Sciences, UCL/ISV, University of Louvain, Croix du Sud 4, L7.07.14, 1348 Louvain-la-Neuve, Belgium

**Keywords:** autophagy, autophagosome, plant cells, Arabidopsis, tobacco, autophagy assays, methods

## Abstract

Autophagy is a catabolic process used by eukaryotic cells to maintain or restore cellular and organismal homeostasis. A better understanding of autophagy in plant biology could lead to an improvement of the recycling processes of plant cells and thus contribute, for example, towards reducing the negative ecological consequences of nitrogen-based fertilizers in agriculture. It may also help to optimize plant adaptation to adverse biotic and abiotic conditions through appropriate plant breeding or genetic engineering to incorporate useful traits in relation to this catabolic pathway. In this review, we describe useful protocols for studying autophagy in the plant cell, taking into account some specificities of the plant model.

## 1. Introduction

When embarking into the field of plant autophagy, a plant cell biologist may rely on the many reviews that have flourished within the last decade, which propose many valuable tools to investigate and monitor autophagy in plant cells [1,2,3,4,5,6,7,8,9,10,11]. The experimenter is also encouraged to refer to the “Guidelines for the use and interpretation of assays for monitoring autophagy” [12], which constitutes a rich source of references to embrace methods for studying autophagy in eukaryotic cells, and proposes a glossary dedicated to autophagy-related acronyms. It would be impossible and even counter-productive to detail all the available approaches found in the literature quoted above. In this review, we aim at providing an overview of the basic assays used in plant cell biology to address the “understanding of plant macroautophagy”. After a brief reminder of the plant specificities, which have to be taken into account when studying autophagy processes in plant cells, the selection of protocols reported herein will be presented for their easiness and/or reliability and/or robustness. We will focus mainly on one plant species, namely Arabidopsis including its cell culture derivatives, but will also discuss complementary approaches in the tobacco plant and the derived BY2 cultured cells, and will share some cautionary limitations based on the literature and our personal observations.

## 2. Peculiarities of the Plant System Regarding Autophagy Processes

Autophagy is a catabolic process deemed to maintain or restore cellular and organismal homeostasis. Technological developments have paved the way towards the understanding of autophagy. The reader is referred to the lecture by Yoshinori Ohsumi, Nobel Prize in Physiology or Medicine in 2016, for the discovery of genes essential for autophagy (https://www.nobelprize.org/nobel_prizes/medicine/laureates/2016/ohsumi-lecture.html). Autophagy can be described as a cellular process permitting the sequestration of a portion of the cytoplasm within a defined membrane compartment, and its transport to a lytic compartment for degradation into macromolecule building blocks, which can be recycled when needed. In addition to acting as a nutrient recycling process important during starvation, this pathway can also get rid of dysfunctional organelles, physiologically detrimental macromolecules, or even intracellular pathogens. In plants, basal (or constitutive) autophagy is essential for plant growth and development. Moreover, similar to what has been described in other eukaryotes, plant cells use, regulate, and activate this process to overcome physiological insults linked to biotic or abiotic stresses [3].

**Figure 1:**
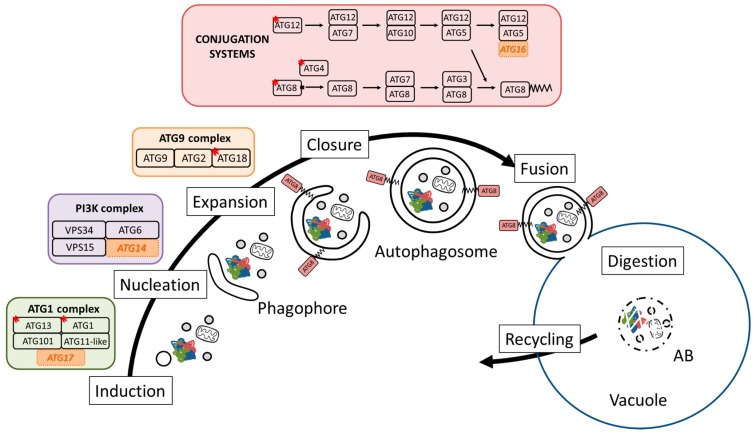
Conservation of the core autophagy machinery in *Arabidopsis thaliana*, represented by the four main complexes acting along the lifecycle of an autophagosome. (*) indicates the occurrence of several isoforms of the protein in plants. *Italics* in orange boxes indicate the absence of a functional counterpart in *Arabidopsis thaliana*.

Autophagy is a complex and highly regulated process. At least two types of autophagy have been described in plant cells: microautophagy and macroautophagy. Both microautophagy and macroautophagy can be non-selective (bulk autophagy) or selective. Microautophagy, during which a portion of the cytoplasm is directly engulfed by invagination of the vacuolar membrane is, however, still mechanistically poorly understood in plant cells [7]. Macroautophagy (hereafter simply referred to as autophagy) is the most studied autophagy process and is described in several experimental plant models [1,11,13,14]. Macroautophagy is characterized by de novo-generated double-membrane compartments (the autophagosomes) that sequester portions of the cytoplasm. The outer membrane of the autophagosome then fuses with the tonoplast, delivering its content as a single membrane-bound compartment (or autophagic body) into the vacuolar lumen (Figure 1).

Plants have the specificity to rely on photosynthesis for their carbon source; therefore, any environmental variation that may detrimentally affect photosynthesis can be translated as carbon deprivation and induces plant autophagy signaling and regulation. Nitrogen is also a key nutrient for plant growth and has to be acquired from the soil and translocated into the plant organs for metabolism. Nutrient starvation through nitrogen or carbon deprivation is a strong inducer of non-selective macroautophagy, triggering the recycling of many soluble proteins to provide nitrogen and carbon sources to face the starvation. This strategy increases the turnover rate of the proteins and allows the plant to rapidly adjust its metabolism as physiologically needed and to ensure its development [15].

In contrast, selective autophagy involves defined receptors interacting with their cognate cargo and the autophagy machinery to trigger their degradation through the autophagic pathway. This selective pathway targets specific cytoplasmic components (organelles down to specific macromolecules) and contributes to cellular protection through the sequestration and degradation of damaged or dysfunctional intracellular components and organelles. Considering the diversity and specificity of cargo degraded by this pathway, there may be plant-specific selective autophagy. A good example of plant selective autophagy is chlorophagy, characterized by whole photodamaged chloroplasts being selectively targeted for degradation, thereby limiting the accumulation of reactive oxygen species in the cell ([16], see also [12] for more references). Other types of selective autophagy active in plants include proteaphagy [17], intraplastidial autophagy [18], and mitophagy [19,20]. The variety of plant-specific cargo, their size, and their complex subcellular localization hint at the diversity in the initiation mechanism and the final size of autophagosomes in plants.

In addition, studying the dynamics of autophagosomes in plant cells present some technical challenges as the cytoplasm represents a highly restricted space, notwithstanding that the compartmentation of the plant cell differs slightly from the one reported in yeast and mammals. The plant cells’ vacuoles are highly pleomorphic compartments, changing in shape, function, and contents according to cell development and growth stage [21], suggesting fine interactions with the autophagic processes that are known to modulate the homeostasis of the plant vacuolar membrane. Another plant-specific characteristic is the apparent ability to perform direct fusion of the autophagosomes with the lytic vacuoles, without the occurrence of intermediate lytic compartments generated by the fusion of autophagosomes with some pre-lysosomal compartments usually described in mammalian cells [9,10,11,22,23]. In all cases, the autophagy process is marshalled by the sequential involvement of defined and evolutionary conserved AuTophaGy-related (ATGs) proteins. The core autophagy machinery initially described in yeast is well conserved in the animal and plant kingdoms. However, defined mechanistic components are intriguingly missing in known plant genomes (Figure 1). These include a genuine homolog of the scaffold subunit RB1CC1/FIP200/ATG17 of the ATG1 complex. The characterization of an ATG11-like plant protein has been described and it is functionally involved in selective autophagy [19,20]. Whether this ATG11-like protein containing both ATG11 and ATG17-related domains acts as a bifunctional scaffolding protein within the ATG1 complex in plants is not yet known. Similarly, a plant functional homolog of ATG14, a component of the class III PI3P kinase complex, has yet to be identified. Based on sequence homologies, a homolog of ATG16L (a component of the ATG12/ATG5) also appears to be missing in plants. The Arabidopsis ATG5G50230 gene may code for a potential yeast ATG16 homolog, but this gene or any plant homolog has not been functionally characterized. Plant ATG9 proteins, with only one isoform in plants, appear to be structurally different from yeast and animal ATG9, with a characteristic very long C-terminal extension among other features [24]. In addition, some of the core ATGs are exceptionally diversified in plants with no clear indication so far of functional divergence or convergence of the various isoforms (Figure 1). Despite these noticeable differences, autophagosome formation and maturation in plants appear to require the same highly regulated sequential steps described in other kingdoms.

## 3. Plant Experimental Models: Whole Plant Versus Cell Cultures

Many plant species including crops have their genomes sequenced. Most of these sequenced species are amenable to genetic transformation. A literature survey shows that Arabidopsis, rice, tobacco, and *Chlamydomonas* have been preferentially used as plant experimental models to study autophagic processes. *Chlamydomonas* has the advantage of having single copy genes of the ATG machinery [12,25]. However, *Arabidopsis thaliana* remains a favored model because of its well-characterized genetics, the availability of functional genomics tools, and the feasibility of transient expression of any gene of interest [26] or stable expression using established protocols for this species. Whole plant gametophyte can be transformed by *Agrobacterium*-mediated T-DNA transfer and the resulting transgenic seedlings can be observed directly under the objective of a light microscope without further invasive preparation.

**Figure 2:**
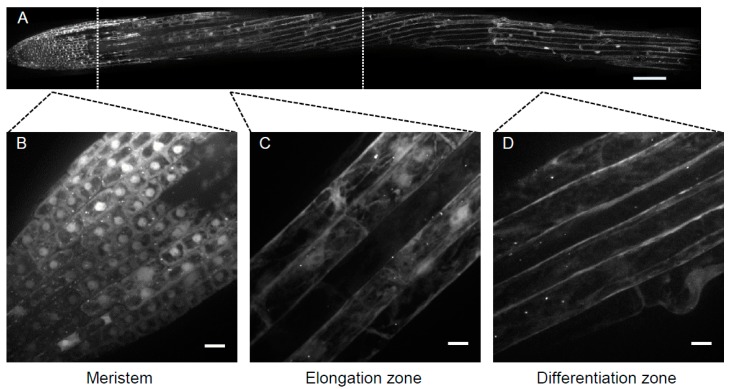
(**A**–**D**) Longitudinal distribution of the autophagy marker ATG8 linked to the green fluorescent protein (GFP) in Arabidopsis in the elongation zone (**C**) and differentiation zone (**D**). Images represent maximum intensity root (see Section 5 and Section 7). (**A**) Expression of GFP-ATG8f in 5-day-old Arabidopsis root in the whole root (**A**), corresponding to three distinct physiological zones (separated by dotted lines), and zoomed in (**B**–**D**): note the ATG8-labeled fluorescent structures in the meristematic zone (**B**) but the low level of expression projection (MIP) of a Z-stack within 60 µm of tissue thickness. Scale bar: (**A**) 100 µm; (**B**–**D**) 10 µm.

For light microscopy, most of the analyses are commonly focused on root cells to avoid the strong autofluorescence from the chloroplasts in green tissues. In the root, focus is especially on epidermal tissues because they are more accessible. It is very important to conduct the experiments on the same region as relative autophagy levels can vary substantially along the root (Figure 2).

**Figure 3:**
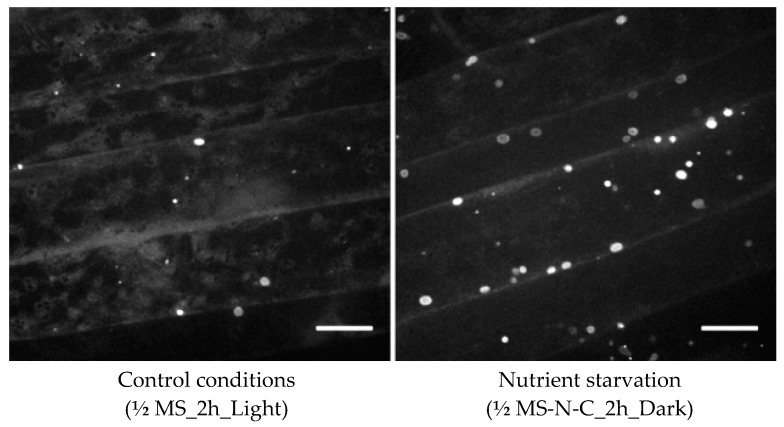
Induction of autophagy by nutrient starvation in the differentiation zone of Arabidopsis root expressing GFP-ATG8. (**Left**) Control conditions with the occurrence of rare GFP-ATG8-labeled structures; (**Right**) after nutrient starvation, an increase in GFP-ATG8-labeled structures after 2 h in a nitrogen- and carbon-deprived medium. Spinning disk microscopy maximum intensity projection (MIP) of 20 µm stacks. Scale bar = 10 µm.

For instance, in the root tip, the basal autophagy level is relatively high and constant, while in the root differentiation zone the basal level of autophagy is very low but increases strongly upon induction [22]. Therefore, when specifically working on autophagy induced by starvation, analysis of the differentiation zone will be favored (Figure 3). It should be reminded that the fluorescent signals observed are actually a snapshot of a fine balance between the rate of autophagosome formation and the rate of autophagosome degradation in the vacuole. Thus, the use of fluorescent signals associated with autophagy markers to define the relative activity of autophagy could be misleading as it may correspond either to an increase of autophagosome formation or to a blockage of autophagosome fusion with the vacuole. Observations of an increase of marker signals should, therefore, be completed by checking the rate of degradation upon inhibition of vacuole degradation of the autophagosomes (see the “concanamycin test”, protocol 5 below).

For biochemical approaches, the small size of the Arabidopsis plant is a limiting factor. For differentiated tissue response analyses, the best alternative is tobacco. Many tobacco species such as *Nicotiana tabacum* and *N. benthamiana* are easily transformable through *Agrobacterium*-mediated T-DNA transfer, generating transgenic lines with relatively higher biomass compared to Arabidopsis. Undifferentiated cell cultures may also be of interest for biochemical approaches. Arabidopsis cell suspension culture [27] and Bright Yellow-2 (BY-2) derived from *N. tabacum* are commonly used [28,29]. However, differences in autophagy responses have been reported between suspension cell cultures and whole plants, such as the occurrence of intermediate compartments between autophagosomes and vacuoles, named autolysosomes [27,28]. It is worth stressing that only the integration of experimental data from different experimental models can allow the distinction between core signal transduction cascades regulating autophagy response in plant and the evolution of plant-specific strategies, or tissue specificities underlying adaptation to new physiological requirements. Table 1 summarizes the pros and cons of some common plant models that could be used as experimental systems.

## 4. Phenotypic Characterization of Plant Autophagy-Deficient Mutants

Loss-of-function *ATG* gene mutants are available from different plant species, and the resulting plant phenotype may serve as a guide to specify the function of a given ATG protein in the autophagy process. However, most of the described ATG mutant lines have only shown subtle if any visible phenotype as compared to wild-type plants under normal growing conditions. This could be explained in part by functional redundancy within the multigene family encoding some of the autophagy core genes in plants, although mutations in single genes encoding loci such as ATG5 and ATG9 also show no major phenotype under normal growth conditions. Noticeable exceptions are *atg6* and *vps34* null mutants, which are embryo-lethal [30]. In aging Arabidopsis shoots, autophagy-deficient mutants display a premature senescence of the leaf tissues and, interestingly, also the siliques, suggesting a possible impact in seed ripening. The relative hypersensitivity to abiotic stress or to nutrient deficiency of autophagy-deficient plants, as compared to the wild-type plant, is a commonly described macroscopic observation, irrespective of the species or the gene affected. However, the described visible symptoms (chlorosis, early senescence, or growth defect) are not specific enough to be ascribed to autophagy deficiency alone. In addition, the severity of the phenotype varies with the gene affected, and most of the time the protocol used to highlight the role of an ATG protein has to be specifically adjusted. Furthermore, other affected pathways unrelated to autophagy could generate similar symptoms under identical growth conditions. Therefore, a great deal of relevant and informative approaches to identify and analyze autophagy in plants relies essentially on cell biology approaches.

## 5. Cytochemistry of Plant Autophagy

Many dyes have been used to label autophagosomes in mammals and some of them have also been tested in plants. The great majority of these markers rely on the presumed acidity of the autophagosome for accumulation of the dye. However, when used in plant cells, disappointment is often “*au rendezvous*”, as these dyes do not usually give clear-cut results. Notwithstanding the fact that autophagosomes’ pH has never been measured in plant cells, non-autophagosomal plant lytic compartments are also detected by these probes. Therefore, the identity of small compartments labeled by dyes such as neutral red, quinacrine monodansylcadaverine, Cyto ID autophagy detection kit (Enzo Life Sciences (ELS), Villeurbanne, France), and Lysotracker Red DND-99 (Life Technologies SAS, Villebon-sur-Yvette, France) or similar products may not be limited to autophagosomal structures. Most of the time such dyes may even fail to label autophagosomes. The recent development of fluorescent protein-based pH sensors expressed simultaneously with ATG8 fluorescent fusion could be used to monitor the relative pH of plant autophagosomes in different tissues and under physiological conditions [31]. However, the unique profile of autophagosome formation and the short life of true autophagosomal structures may constitute a real technical challenge. In cultured cells, the induction of autophagy by starvation triggers the formation of numerous acidic vesicles [27,29]. These vesicular compartments accumulate further in the presence of protease inhibitors and are stained with acidic dyes such as neutral red and Lysotracker. However, mCherry-ATG8 was never found colocalizing with those stained structures, suggesting that the probes were not specific for autophagosomes (Raulin and Le Bars, personal communication). Contrastingly, in other experimental models, the autofluorescent compound monodansylcadaverine (MDC) has been reported to be associated with ATG8-labeled autophagic vacuoles [5]. In effect, these results outline the fact that the use of such dyes has to be performed with care in plant cells.

## 6. Live Imaging of Autophagy in Plants

**Figure 4:**
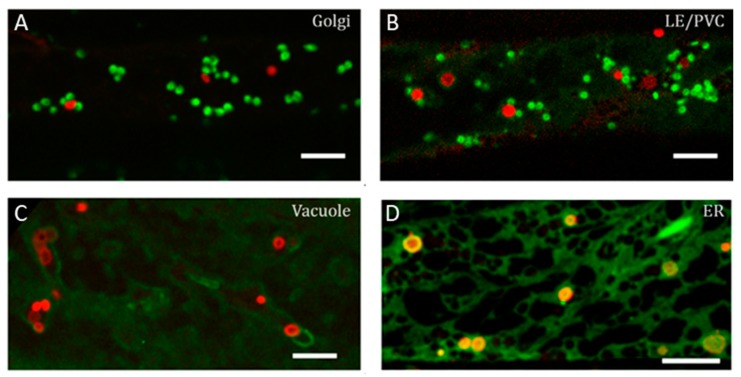
Example of colocalization investigation between the autophagosomes and other endomembrane markers using stable co-expression in *Arabidopsis thaliana*. F1 plants resulting from the crossing between Arabidopsis lines stably expressing Yellow Fluorescent Protein (YFP) tagged endomembrane markers (green) with a line stably expressing mCherry-ATG8e (red). (**A**) Autophagosome membranes do not show clear colocalization with the Golgi (SYP32); (**B**) nor late endosomes/prevacuolar compartments (LE/PVC, Rab-G3f) or (**C**) vacuoles (VAMP711), with the red signals (autophagosomes) being distinct from the green signals (Golgi, prevacuolar compartments, tonoplast, respectively); (**D**) Interactions with the endoplasmic reticulum (ER) (labeled by a GFP-HDEL fusion) is indicated by the orange color given by the superposition of the green (ER) and red (autophagosomes) signals. Scale bars: 5 µm.

Fluorescence microscopy remains the most used approach for detecting autophagy in plant cells [5]. Expression of ATG proteins fused to a fluorescent reporter appears to be a far more reliable tool than fluorescent dyes to investigate the dynamics of autophagosomes in living plant cells. ATG8 is thought to be a useful marker to monitor autophagy stages, as it is detectable right from the initiation stage up to the degradation of the autophagosome in the vacuole. Nevertheless, it is important to keep in mind that only the N-terminal fusions of ATG8 can be used because of the processing of the C-terminal part of ATG8, followed by the enzymatic lipidation and anchoring to the phagophore membranes. When visualizing the expression of such fusion chimera, we have to bear in mind that the lipidated form of the fluorescent chimeric ATG8 will be associated with the autophagosomal membranes, whereas the non-lipidated form will remain cytosolic and generate a high background signal that can perturb or mask the detection of ATG8-positive puncta. The fluorescent proteins used to label the ATG proteins have to be chosen carefully, especially because of their behavior in acidic environments. For instance, when focusing on imaging autophagic bodies within the vacuole, one has to be aware that the fluorescent protein requires a p*K*_a_ lower than the vacuolar pH. In this case, the use of the mCherry variant (p*K*_a_ < 4.5) is a better choice than the green or yellow fluorescent variants. In comparison, the Green Fluorescent protein (GFP) (p*K*_a_ ~ 6.0) will be quenched in such environments. GFP-ATG8 expression in root epidermal cells of the differentiation zone can be observed in control conditions as rare puncta moving with the cytosolic streaming (Figure 2). Induction of autophagy by nutrient starvation (protocol 4 described below (see Section 7) is accompanied by an important increase in ATG8-labeled structures and also an increase of their size [22] (Figure 3). Transgenic plant lines expressing ATG protein markers in a stable manner may be combined with other markers of membrane compartments [32] to perform combinatorial analyses in order to investigate potential spatial interactions between ATG proteins and markers of the endomembrane systems (Figure 4).

A reasonable number of fluorescent protein-tagged ATG fusion chimera have now been tested in the plant autophagy field, and their functionality is a prerequisite for strengthening the interpretation of the resulting data. This is not always possible or straightforward when the ATG protein considered is essential. However, a lack of functional demonstration of the chimeric fusion could lead to artifacts in terms of subcellular localization and dynamics.

Multiscale imaging of biological processes in plant cells has been discussed in recent reviews [33,34]. However, at the tissue and cellular levels, the detection of fluorescently-tagged ATG proteins can theoretically be achieved on any wide-field fluorescence microscope. However, when observing whole plant tissues, detection of the resulting dim punctate signals can prove to be very challenging. A first common limitation is autofluorescence in plant cells and the diffuse fluorescence surrounding the focal plane. A second limitation is the size of the object of interest, which can be smaller than the resolution limit of a standard fluorescent microscope (lateral resolution: 350 nm). The use of confocal laser scanning microscopy (CLSM) can circumvent those difficulties by detecting only the photons emitted at the focal plane, thus increasing the contrast and the resolution of the images (250 nm lateral resolution). However, when working with living tissues, the scanning process is too slow (approximately 1 frame/s) for it to be compatible with image acquisition of autophagosomes that are moving at a velocity higher than 5 µm·s^−1^. Moreover, the illumination process of CLSM may provoke bleaching of the specimen, preventing the observation of long-term processes. It may also generate an additional stress, inducing non-controllable autophagic responses.

The best compromise between resolution, phototoxicity, and frame rate is the use of a spinning disk microscope, which will allow the capture of dynamic events (up to 30 images per second achievable on biological samples) with a confocal resolution. Protocol 1 provides information on the setup currently used in our laboratory. Using spinning disk microscopy, the maximal observable thickness is smaller than in CLSM but in our hands fluorescent protein-tagged ATG such as ATG5 signals can be easily recorded within 2–3 of the most external cell layers of the Arabidopsis differentiated root. A maximal projection (MIP) of the various optical sections allows 2D mapping of the ATG-labeled structures, while a 3D analysis of the data allows robust measurement of the size and number of structures per cell [22]. A similar approach was recently used to investigate the dynamics of plant ATG9-labeled structures [24]. The development of imaging solutions and image analysis tools are becoming essential for comprehending the autophagy process in plants.

**Protocol 1: An imaging setup for live imaging of plant cells**
 Confocal images are acquired with a Nipkow spinning disk confocal system (Yokogawa CSU-X1-A1, distributed by Roper scientific, Lisses, France) mounted on a Nikon Eclipse Ti E inverted microscope, equipped with a 100× Apochromat TIRF oil-immersion objective (NA: 1.49). Blue (491 nm CoBolt Calypso DPSS, 100 mW) and yellow (561 nm Cobolt Jive DPSS, 150 mW) lasers are used for excitation of YFP and mCherry, respectively. A dual-band dichroic mirror (491/561 nm Chroma distributed by Roper Scientific, Evry, France) is used and band-pass filters of 525/45 nm and 607/36 nm (Semrock, distributed by Roper Scientific, Evry, France) allows the detection of YFP and mCherry, respectively. Images are recorded with an EMCCD Evolve camera (Photometrics, distributed by Roper Scientific, Evry, France). The whole system is driven by Metamorph software version 7.7 (Molecular Devices distributed by Roper Scientific, Evry, France).

It is worth stressing that manipulating the seedlings under the microscope may also induce autophagy, probably because of the mechanical pressure of the coverslip or the rarefying oxygen in the medium [35]. To minimize such adverse and uncontrollable stresses, the use of a microfluidic chip device is recommended [36,37] when conducting these experiments in roots. Using this device, one can perform long time live imaging of multiple seedlings in parallel in multiple chambers with an individual control of the environment (fresh medium perfused or drug treatments) without inducing autophagy in control conditions.

## 7. Ultrastructural Observations of Autophagosome Formation and Degradation by Electron Microscopy of Plant Samples

Transmission electron microscopy (TEM) has been the first approach to allow the identification of the autophagy process [38] and serves today to describe multiple aspects of autophagy.

TEM has allowed the description of the four main stages along the autophagic pathway (i.e., nucleation of the phagophore, elongation of the phagophore, closure to form an autophagosome, and release of autophagic bodies after fusion of the autophagosome with the vacuole). TEM can also provide information on the spatial distribution of ATG proteins and their subcellular potential interactions.

**Figure 5:**
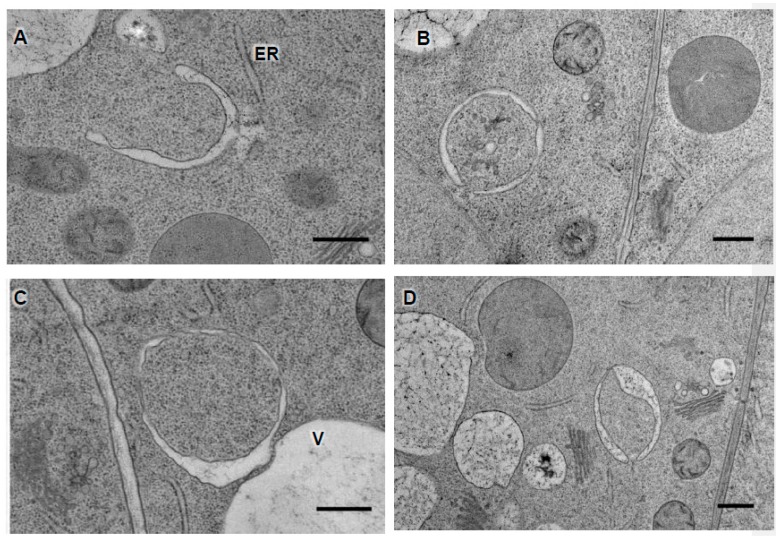
Growing phagophore and mature autophagosomal structures in *Arabidopsis thaliana* (**A**) Growing phagophore, close to the endoplasmic reticulum (ER); (**B**) sequestration of cytoplasmic components in a closing autophagosomal structure; (**C**) note the occurrence of a large space between the two membranes of the autophagosomes that may appear as a result of the sample processing for Electron Microscopy (EM); (**D**) autophagosome with membrane contact site with the vacuole (V). Scale = 500 nm. Root tips are prepared as described in Protocol 2, cryofixed, and embedded in EPON resin. Ultrathin sections (80 nm) were contrasted with uranyl acetate and lead citrate and observed by TEM at 120 kV.

As shown in Figure 5, the most striking feature of a mature autophagosome is the double membrane surrounding a portion of cytoplasm. Also characteristic is the absence of ribosomes at the cytosolic side of the membranes, and a luminal intensity similar to the one present in the nearby cytosol. Visible organelles or parts of organelles may also be seen in the lumen of the autophagosome. However, contrasting with other double-membrane bounded organelles such as chloroplasts, mitochondria, or even the nucleus, the space between the double membranes of autophagosomes appears to be filled with material highly sensitive to the processing protocols for electron microscopy. It is therefore not unusual to observe a large empty gap between the membranes, often pointed as fixation artifacts and outlining probably some extractable components. Various protocols have been tested to optimize the ultrastructural aspect of autophagosomes. Conventional embedding protocols in epoxy resins for ultrastructural observations or acrylic resins for immunocytochemistry approaches [39,40] may provide a good deal of information. To optimize the preservation of the autophagosomal structure, we currently favor fixation by high-pressure freezing, which increases the quality of fixation as also described in [41].

**Figure 6:**
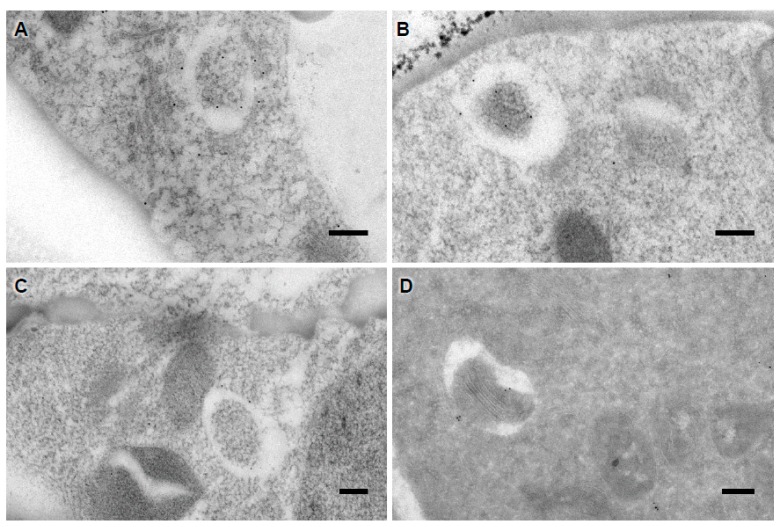
Immunolocalization of ATG8 protein. (**A**–**C**) Plants expressing a GFP-ATG8 construct were included in glycol methanlacrylate acrylic (GMA) resin as described in [40]. Then, 80 nm sections were collected on a nickel grid and immunolabeling was performed with an antibody against GFP; (**D**) Cryosections of Arabidopsis roots expressing GFP-ATG8 and decorated using anti-GFP antibodies. Scale bars: 5 µm.

Protocols to reveal the spatial distribution of endogenous ATG proteins or their interactions with intracellular components may also be achieved at the Electron Microscopy (EM) level by an immunogold labeling technique (Protocol 3). However, it may be a high challenge because of the relative scarcity of some ATG proteins, the variation of autophagic response within root tissues, and the transient lifetime of ATG proteins and autophagosomes, not to mention the difficulty of having high-quality isoform antibodies. The use of anti-GFP on sections issued from plants expressing a functional fluorescent ATG reporter may appear as a better option (Figure 6). The experimenter could favor immunogold on LR White sections [39], Lowicryl HM20 sections [40], or on cryosections using the Tokuyasu method [22,42]. The use of glycol methanlacrylate acrylic (GMA) resin has proven to be an excellent alternative but it is far more difficult to work with due to its fragility when sectioning [43].

**Protocol 2: Ultrastructural observation of autophagosome in the plant cell**
 Three-millimeter root tips are cut in 1-hexadecen then transferred to 200 µm-sized cupules (Leica, Ref. n° 16706897) containing 1-hexadecen and frozen with a high-pressure freezer apparatus (EMPACT2, Leica France).  Freeze substitution is carried out in a Leica freeze substitution unit (AFS2, Leica France), in acetone supplemented with 2% osmium tetroxide warming up progressively from −90 °C to −30 °C (specimens are left at −90 °C for 27 h, then warmed up to −60 °C over 15 h; specimens stay in a −60 °C bath for 8 h before the next warmup step to −30 °C over 15 h, where they remain for an additional 8 h).  Root tips are then infiltrated and embedded in epoxy resin (low viscosity premix kit medium, Agar Scientific, Gometz-la-Ville, France) according to the manufacturer’s instructions at room temperature. For polymerization, they are placed in flat plastic molds and polymerized for 17 h at 60 °C.  Then, 80 nm ultrathin sections (Ultracut UC6, Leica) are collected on formvar-coated copper grids. They are post-stained with aqueous 2% uranyl acetate/lead citrate as described in [39]. They are examined with a JEOL 1400 transmission electron microscope (JEOL (Europe), Croissy-sur-seine, France) operating at 120 kV. Images are acquired using a post-column high resolution (11 megapixels) high-speed camera (SC1000 Orius, GATAN France, Evry, France).

**Protocol 3: Immunolocalization of ATG protein by the Tokuyasu method**
 Seedlings are fixed for 1 h with 3% paraformaldehyde (PFA), 1% glutaraldehyde in 0.1 M sodium cacodylate, pH6.8. After three rinses in cacodylate buffer, seedlings are incubated for 10 min in 50 mM glycine and rinsed three times with cacodylate buffer. A drop of 12% gelatin is put on a microscopic slide, the seedlings are immersed in the drop, and a coverslip is applied on the drop to compress the gelatin.  A small block of gelatin containing the root tip is cut with a razor blade and immersed in 80% sucrose overnight at 4 °C. The gelatin block is placed on specimen carriers for cryosectioning (Leica Microsystems, ref. n° 16701950) and plunged in liquid nitrogen. Cryosectioning is performed at −100 °C. Then, 80 nm sections are collected on a nickel grid in a 2% methylcellulose: 80% sucrose drop (1:1 *v*:*v*).  After four baths of PBS, four baths of PBS–glycine100 mM, and two baths of PBS–BSA acetylated, immunolabeling is performed using antibodies against GFP (dilution 1/10) (Abcam, Paris, France, Ref. n° Ab6556). The antigen is visualized through the use of an anti-rabbit (dilution 1/20) conjugated to 10 nm gold particles (AURION distributed by LFG Distribution, Tassin La demi Lune, France).

Finally, the best way to claim that a fluorescent structure is indeed an autophagosome is to perform correlative approaches to integrate multiscale levels, and to associate a fluorescent signal to a well-known structural support. We have developed correlative approaches based on the recognition of the same region of interest by light and electron microscopy, comparing different protocols and their limits [43].

## 8. Experimental Tools to Induce Autophagy in Plants

As previously mentioned, autophagy is a constitutive process occurring under normal growth conditions, but at very low rates. To study the formation of autophagosome and their dynamics, it is recommended to set up the right physiological conditions to amplify the autophagy process and obtain an autophagic response suitable for subsequent analysis. Autophagy is enhanced or triggered in the case of nutrient deficiencies, abiotic stress, or tissue senescence. Senescence is intimately associated with upregulation of autophagy processes, as senescent leaves and cotyledons need to remobilize carbon and nitrogen efficiently for survival. Growing plants in the dark for several days easily achieves carbon deprivation and, therefore, this particular treatment is used to assess the phenotype of autophagic mutant plants, which should exhibit early symptoms of senescence under these conditions.

In laboratory settings, induction of autophagy by nitrogen deprivation and/or carbon deprivation is commonly used in most studies. It requires a synthetic growth medium (Protocol 4). A literature survey shows quite important variations in the protocols of autophagy induction, which may lead to substantial differences in data interpretation, also suggesting that the burst of autophagic response may be spatially and temporally regulated [22,23,44]. For instance, the description of autophagosomes and autophagic flow performed after 48 h of growth in deprived medium may mirror biological processes distinct from a primary response to starvation observed after two hours of starvation. It is essential, therefore, to conduct a time-course experiment in order to integrate the time-dependent dynamics of the process and the relevant interpretation. Using Protocol 4 described below, the burst of autophagy materialized by an increased number of autophagosomes (as observed in Figure 2) was generally observed about 2 h after induction.

**Protocol 4: Induction of autophagy in Arabidopsis roots by nutrient starvation**-Arabidopsis seedlings expressing GFP-ATG8 are grown on a complete synthetic medium (Murashige and Skoog (MS) medium with 1% sucrose as the carbon source).-Five-day-old seedlings are transferred into Petri dishes containing half-strength Murashige and Skoog liquid medium, depleted of nitrogen and carbon: Murashige and Skoog micronutrient salts (Sigma-Aldrich, Lyon, France, Ref. n° M0529), 3 mM CaCl_2_, 1.5 MgSO_4_, 5 mM KCl, 1.25 mM KH_2_PO_4_, 0.5% (*w*/*v*) mannitol, 3 mM 2-(*N*-morpholino) ethanesulfonic acid, pH 5.6; the seedlings are incubated in the dark with gentle rotatory shaking at 100 rpm (nutrient starvation conditions).-Seedlings are mounted between the glass slide and coverslip and immediately observed under the microscope. To avoid autophagy induction under the microscope, the observation should not be longer than 15 min with the same slide.

Pharmacological treatments can also be used to induce autophagy in plants (Table 2), but we believe that all these compounds should be manipulated with care as they may affect other signaling pathways within the plant cell. Indeed, most of these compounds have been thoroughly assessed for off-target effects in plants during all developmental stages. Another important question is the specific type of autophagy induced by a given compound and the related interpretation of the resulting findings. For some of these compounds, the mechanisms of their action in the plant cell are not yet clear.

## 9. Experimental Tools to Inhibit Autophagy in the Plant Cell

Experimental manipulation of the autophagy pathway may help to decipher the mechanisms involved in the formation and dynamics of autophagosomes. The use of ATG mutants has contributed in identifying the underlying molecular machinery involved in the biology of autophagosome formation. Functional redundancy or specificity within otherwise multigenic ATG protein families as encountered in plants could, however, complexify the use of this approach. The use of chemicals with specific targets can overcome functional redundancy and help probe the function of a given ATG protein [54].

Chemical agents (Table 1) have been used to inhibit specific steps of the autophagosome formation in plant cells. For instance, any drug targeting the plant target of rapamycin (TOR) complex signaling should affect the autophagic pathway, although rapamycin appears not to be that effective in inducing autophagy in the plant cell. Reactive Oxygen Species (ROS)-producing agents such as methyl viologen, or inhibitors of phosphatidylinositol 3-kinases such as 3-methyladenine and wortmannin, inhibit autophagosome formation in plants. It is worth stressing that 3-methyladenine, wortmannin, and for that matter LY294002, are not autophagy-specific drugs as they do affect other aspects of membrane trafficking or the structure-function of other endomembrane compartments in the plant cell ([11,12] and literature therein). Regarding the degradation of the formed autophagosome in the lytic vacuole, the membrane-permeable cysteine proteases E-64d and E-64c are potent inhibitors of this event, resulting in “autolysosome” accumulation in some experimental models [27,28,55].

## 10. “The Concanamycin Test”: A Basic Protocol to Detect Autophagy Flux in the Plant Cell

An easy assay to detect and measure autophagy flux (or to assess the impact of a given mutation on autophagy flux) is to apply what has been coined the “concanamycin test”. This test can also be used to check for the restoration of autophagy in a mutant plant complemented with the missing protein [22].

**Figure 7:**
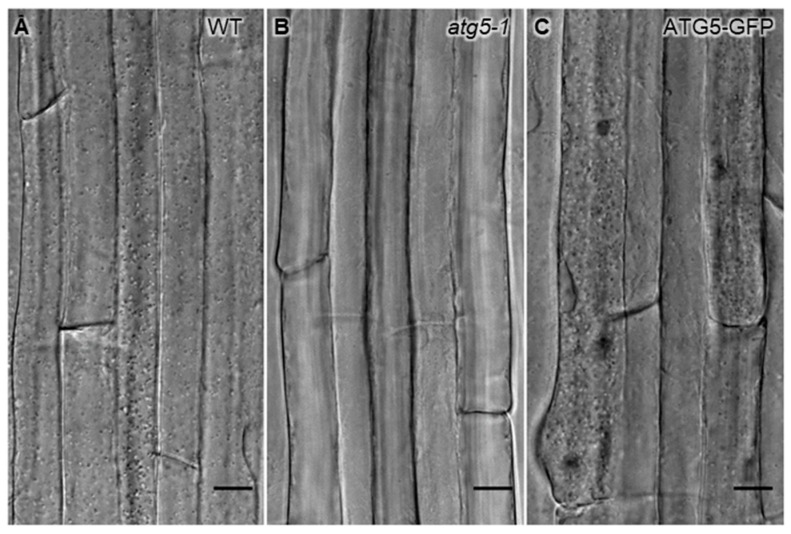
Expression of ATG5-GFP restores accumulation of autophagic bodies in *atg5-1* plants. Differential Interference Contrast (DIC) micrographs of root epidermal cells incubated for 8 h with 1 µM concanamycin A in nutrient starvation conditions. (**A**) Autophagic body accumulation in the vacuole is clearly visible in wild-type (WT) seedlings, (**B**) but absent in the *atg5-1* mutant, (**C**) and restored in *atg5-1* seedlings expressing ATG5-GFP. Scale bar = 15 µm.

Concanamycin A (ConcA) inhibits the vacuolar proton pump V-ATPase, therefore affecting the activity of the vacuolar proteases and hydrolases and preventing the degradation of autophagosomes within the vacuolar lumen. ConcA treatment induces characteristically the accumulation of autophagic bodies within the vacuole. In plant cells in particular, the “autophagic bodies” are easily detectable either by Nomarski differential interference contrast (Figure 7) or by confocal microscopy if the cells are expressing a fluorescent reporter protein (see also Figure 1 in [22]).
**Protocol 5: The “concanamycin test”**
 A 1 mM concanamycin stock solution in DMSO is diluted to 1 µM in either (A) normal culture medium ½ Murashige and Skoog (MS) or (B) medium depleted in nitrogen and carbon. Ten plantlets are immersed in 2 mL of the concanamycin treating solution for 8 h in 55 mm Petri dish and agitated gently (50 rpm) on an orbital shaker in the dark. Petri dishes are coated with Sigmacote (Sigma-Aldrich, Lyon, France, Ref. n° SL2,) to prevent any interaction of the drug with the plastic that could decrease the drug’s efficiency.

## 11. Concluding Remarks

Multiscale imaging combined with the use of the molecular biology toolbox is essential for studying the autophagic pathway in plant cells. Recent technological developments for imaging plant cells have opened the way for gaining further information on detailed biological process together with solid statistical quantitative observations. For instance, biosensor imaging is a forefront tool to decipher the subtle mechanisms underlying plant biological processes [56]. Engineering of new sensors to allow the detection of dynamic changes in the physiology of plant autophagosomes has yet to come. Furthermore, the emerging field of interactions among the catabolic pathways in cells [57] needs further methodological investments to decipher the coordination of such events.

Future success will critically depend on our ability to combine technological and conceptual advances in different disciplines. For instance, results extracted from image data may be combined with mathematical approaches to construct models that may mirror the dynamics of the system. Such modeling may help to explain or predict the variability of cell size or the timing of autophagy processes among plant cell types. The application of current or newly developed technologies may then meet the quest for understanding some specific aspects of the autophagy process in plants.

## Figures and Tables

**Table 1:** Advantages and disadvantages of some plant species models used to study autophagy.

Species ^(1)^	Cell Type	Observations
Arabidopsis	Root	Amenable to direct light microscopy observations and is devoid of chlorophyll-induced autofluorescence; can be processed for TEM. Most light microscopy observations are limited to the epidermal cells; low biomass can limit biochemical analyses.
	Leaves	Most light microscopy observations are limited to pavement cells; chlorophyll autofluorescence can be problematic; difficult to process for TEM.
	Cultured cells	Non-differentiated cells; can be easily transformed and upscaled for biochemical studies. Their physiology may not necessarily reflect the complexity of the tissues/organs they are derived from.
Tobacco	Root	Amenable to direct light microscopy observations and is devoid of chlorophyll-induced autofluorescence; can be processed for TEM. Only a few cells can be observed using light microscopy.
	Leaves	Highly suitable for light microscopy observation (planar). Can be transiently transformed easily through *Agrobacterium* infiltration; relatively higher biomass can facilitate biochemical analyses. Most light microscopy observations are limited to epidermal cells. TEM observations on a very narrow cytoplasmic band.
	Cultured cells	BY-2 cells are cultured in the dark and therefore are devoid of chlorophyll-derived autofluorescence; are relatively large and can be easily upscaled for biochemical analyses. Their physiology may not necessary recapitulate that of differentiated tissues or whole plant.
*Chlamydomonas*	Unicellular organism	Autophagy gene function studies are easier because of the lack of gene redundancy. The free photosynthetic unicellular physiology may not recapitulate that of a multicellular higher plant.

^(1)^ All these species are amenable to genetic transformation.

**Table 2:** Validated pharmacological compounds modulating autophagy in plants.

Compounds	Working Concentrations	Molecular Target	Molecular Mechanism	Ref.
Bay 11	1–50 µM	Unknown	Inhibits autophagy before autophagosome fusion with the vacuole	[45]
BTH (1) (enzo(1,2,3) thiadiazole-7-carbothioic acid)	50–100 µM	Unknown	Salicylic acid agonist acting as autophagy inducer	[24,46,47]
Concanamycin A	0.5–5 µM	Class III PI3K (Phosphoinositide 3-kinase)	Inhibits autophagy by preventing vacuolar degradation but also affects vesicular transport from the Golgi and the endosome to the vacuole	[24,29,48]
1,4-Dithiotreitol (DTT)	0.5–5 mM	Protein disulfides	Prevents the oxidation of SH (thiol) groups and reduces disulphides to dithiols. Induces unfolded protein response in, and autophagic degradation of, the ER	[13,44,49]
E-64	10–100 µM	Vacuolar proteases	Cysteine protease inhibitor. Inhibits autophagic cargo degradation in the vacuole	[28,50]
LY294002	10–50 µM	Class III PI3K	Inhibits autophagy at the autophagosome initiation and/or expansion stage; affects the prevacuolar compartments	[29]
3-Methyladenine	2–5 mM	Class III PI3K	Inhibits autophagy at the autophagosome initiation and/or expansion stage	[28,50,51]
Rapamycin	1–5 µM	Target of rapamycin (TOR)	Enhances autophagy	[25]
Spautin-1	10–50 µM	USP10 and USP13 (Ubiquitin-Specific Proteases 10 & 13)	Inhibits autophagy by enhancing the proteasomal degradation of the class III PI3K complex	[52]
Trehalose	nd	Unknown	TOR-independent inducer of autophagy	[53]
Tunicamycin	1–5 µg/mL	*N*-glycosylation	Induces autophagy by triggering unfolded protein response	[13,44,49]
Wortmannin	1–5 µM	Class III PI3K	Inhibits autophagy at the autophagosome initiation and/or expansion stage; affects the prevacuolar compartments	[28,48,50]
ZPCK	1–50 µM	Unknown	Inhibits autophagic cargo degradation within the vacuole	[45]

## References

[1] Bassham D.C. (2009). Function and regulation of macroautophagy in plants. Biochim. Biophys. Acta.

[2] Bassham D.C. (2015). Methods for analysis of autophagy in plants. Methods.

[3] Avin-Wittenberg T., Honig A., Galili G. (2012). Variations on a theme: Plant autophagy in comparison to yeast and mammals. Protoplasma.

[4] Shin K.D., Lee H.N., Chung T. (2014). A revised assay for monitoring autophagic flux in *Arabidopsis* thaliana reveals involvement of autophagy-related9 in autophagy. Mol. Cells.

[5] Pu Y., Bassham D.C., Lois L., Matthiesen R. (2016). Detection of Autophagy in Plants by Fluorescence Microscopy. Plant Proteostasis: Methods and Protocols.

[6] Bao Y., Mugume Y., Bassham D.C. (2017). Biochemical Methods to Monitor Autophagic Responses in Plants. Methods Enzymol..

[7] Chanoca A., Kovinich N., Burkel B., Stecha S., Bohorquez-Restrepo A., Ueda T., Eliceiri K.W., Grotewold E., Otegui M.S. (2015). Anthocyanin Vacuolar Inclusions Form by a Microautophagy Mechanism. Plant Cell.

[8] Takatsuka C., Inoue-Aono Y., Moriyasu Y. (2017). Isolation of Autolysosomes from Tobacco BY-2 Cells. Methods Mol. Biol..

[9] Chen L., Li F., Xiao S. (2017). Analysis of Plant Autophagy. Methods Mol. Biol..

[10] Gomez R.E., Joubes J., Valentin N., Batoko H., Satiat-Jeunemaitre B., Bernard A. (2017). Lipids in membrane dynamics during autophagy in plants. J. Exp. Bot..

[11] Soto-Burgos J., Zhuang X., Jiang L., Bassham D.C. (2017). Update on autophagy: Dynamics of autophagosome formation. Plant Physiol..

[12] Klionsky D.J., Abdelmohsen K., Abe A., Abedin M.J., Abeliovich H., Acevedo Arozena A., Adachi H., Adams C.M., Adams P.D., Adeli K. (2016). Guidelines for the use and interpretation of assays for monitoring autophagy (3rd edition). Autophagy.

[13] Liu Y., Bassham D.C. (2012). Autophagy: Pathways for self-eating in plant cells. Annu. Rev. Plant Biol..

[14] Yang X., Bassham D.C. (2015). New Insight into the Mechanism and Function ofAutophagy in Plant Cells. Int. Rev. Cell Mol. Biol..

[15] Masclaux-Daubresse C., Chen Q., Havé M. (2017). Regulation of nutrientrecycling via autophagy. Curr. Opin. Plant Biol..

[16] Izumi M., Hidema J., Wada S., Kondo E., Kurusu T., Kuchitsu K., Makino A., Ishida H. (2015). Establishment of monitoring methods for autophagy in rice reveals autophagic recycling of chloroplasts and root plastids during energy limitation. Plant Physiol..

[17] Marshall R.S., Li F., Gemperline D.C., Book A.J., Vierstra R.D. (2015). Autophagic Degradation of the 26S Proteasome Is Mediated by the Dual ATG8/Ubiquitin Receptor RPN10 in *Arabidopsis*. Mol. Cell.

[18] Parra-Vega V., Corral-Martinez P., Rivas-Sendra A., Segui-Simarro J.M. (2015). Formation and excretion of autophagic plastids (pastolysomes) in *Brassica napus* embryogenic microspores. Front. Plant Sci..

[19] Li F., Vierstra R. (2014). *Arabidopsis* ATG11, a scaffold that links the ATG1-ATG13 kinase complex to general autophagy and selective mitophagy. Autophagy.

[20] Li F., Chung T., Vierstra R.D. (2014). AUTOPHAGY-RELATED11 plays a critical role in general autophagy- and senescence-induced mitophagy in *Arabidopsis*. Plant Cell.

[21] Bolte S., Lanquar V., Soler M.N., Beebo A., Satiat-Jeunemaître B., Bouhidel K., Thomine S. (2011). Distinct lytic vacuolar compartments are embedded inside the protein storage vacuole of dry and germinating *Arabidopsis* thaliana seeds. Plant Cell Physiol..

[22] Le Bars R., Marion J., Le Borgne R., Satiat-Jeunemaitre B., Bianchi M.W. (2014). ATG5 defines a phagophore domain connected to the endoplasmic reticulum during autophagosome formation in plants. Nat. Commun..

[23] Le Bars R., Marion J., Satiat-Jeunemaitre B., Bianchi M.W. (2014). Folding into an autophagosome ATG5 sheds light on how plants do it. Autophagy.

[24] Zhuang X., Chung K.P., Cui Y., Lin W., Gao C., Kang B.H., Jiang L. (2017). ATG9 regulates autophagosome progression from the endoplasmic reticulum in *Arabidopsis*. Proc. Natl. Acad. Sci. USA.

[25] Pérez-Pérez M.E., Florencio F.J., Crespo J.L. (2010). Inhibition of target of rapamycin signaling and stress activate autophagy in *Chlamydomonas* reinhardtii. Plant Physiol..

[26] Marion J., Bach L., Bellec Y., Meyer C., Gissot L., Faure J.D. (2008). Systematic analysis of protein subcellular localization and interaction using high-throughput transient transformation of *Arabidopsis* seedlings. Plant J..

[27] Rose T.L., Bonneau L., Des C., Marty-Mazars D., Marty F. (2006). Starvation-induced expression of autophagy-related genes in *Arabidopsis*. Biol. Cell.

[28] Moriyasu Y., Inoue Y. (2008). Use of proteases inhibitors for detecting autophagy in plants. Methods Enzymol..

[29] Takatsuka C., Inoue Y., Matsuoka K., Moriyasu Y. (2004). 3-methyladenine inhibits autophagy in tobacco culture cells under sucrose starvation conditions. Plant Cell Physiol..

[30] Kim S.H., Kwon C., Lee J.H., Chung T. (2012). Genes for plant autophagy: Functions and interactions. Mol. Cells.

[31] Shen J., Zeng Y., Zhuang X., Sun L., Yao X., Pimpl P., Jiang L. (2013). Organelle pH in the *Arabidopsis* endomembrane system. Mol. Plant.

[32] Geldner N., Dénervaud-Tendon V., Hyman D.L., Mayer U., Stierhof Y.D., Chory J. (2009). Rapid, combinatorial analysis of membrane compartments in intact plants with a multicolor marker set. Plant J..

[33] Rousseau D., Chéné Y., Belin E., Semaan G., Trigui G., Boudehri K., Franconi F., Chapeau-Blondeau F. (2015). Multiscale imaging of plants: Current approaches and challenges. Plant Methods.

[34] Sozzani R., Busch W., Spalding E.P., Benfey P.N. (2014). Advanced imaging techniques for the study of plant growth and development. Trends Plant Sci..

[35] Wang P., Richardson C., Hawes C., Hussey P.J. (2016). *Arabidopsis* NAP1 Regulates the Formation of Autophagosomes. Curr. Biol..

[36] Grossmann G., Meier M., Cartwright H.N., Sosso D., Quake S.R., Ehrhardt D.W., Frommer W.B. (2012). Time-lapse fluorescence imaging of *Arabidopsis* root growth with rapid manipulation of the root environment using the RootChip. J. Vis. Exp..

[37] Grossmann G., Guo W.J., Ehrhardt D.W., Frommer W.B., Sit R.V., Quake S.R., Meier M. (2011). The RootChip: An integrated microfluidic chip for plant science. Plant Cell.

[38] De Duve C., Wattiaux R. (1966). Functions of lysosomes. Annu. Rev. Physiol..

[39] Hawes C., Satiat-Jeunemaitre B., Hawes C., Satiat-Jeunemaitre B. (1981). Plant cell biology. Practical Approach Series.

[40] Zhuang X., Wang H., Lam S.K., Gao C., Wang X., Cai Y., Jiang L. (2013). A BAR-domain protein SH3P2, which binds to phosphatidylinositol 3-phosphate and ATG8, regulates autophagosome formation in *Arabidopsis*. Plant Cell.

[41] Corral-Martinez P., Parra-Vega V., Segui-Simarro J.M. (2013). Novel features of *Brassica napus* embryogenic microspores revealed by high pressure freezing and fresze substitution: Evidence for massive autophagy and excretion-based cytoplasmic streaming. J. Exp. Bot..

[42] Tokuyasu K.T. (1973). A Technique for Ultracryotomy of Cell Suspensions and Tissues. J. Cell Biol..

[43] Marion J., Le Bars R., Satiat-Jeunemaitre B., Boulogne C. (2017). Optimizing CLEM protocols for plants cells: GMA embedding and cryosections as alternatives for preservation of GFP fluorescence in *Arabidopsis* roots. J. Struct. Biol..

[44] Liu Y., Bassham D.C. (2013). Degradation of the endoplasmic reticulum by autophagy in plants. Autophagy.

[45] Mishra P., Dauphinee A.N., Ward C., Sarkar S., Gunawardena A., Manjithaya R. (2017). Discovery of pan autophagy inhibitors through a high-throughput screen highlights macroautophagy as an evolutionarily conserved process across 3 eukaryotic kingdoms. Autophagy.

[46] Munch D., Rodriguez E., Bressendorff S., Park O.K., Hofius D., Petersen M. (2014). Autophagy deficiency leads to accumulation of ubiquitinated proteins, ER stress, and cell death in *Arabidopsis*. Autophagy.

[47] Gao C., Zhuang X., Cui Y., Fu X., He Y., Zhao Q., Zeng Y., Shen J., Luo M., Jiang L. (2015). Dual roles of an *Arabidopsis* ESCRT component FREE1 in regulating vacuolar protein transport and autophagic degradation. Proc. Natl. Acad. Sci. USA.

[48] Vanhee C., Batoko H. (2011). *Arabidopsis* TSPO and porphyrins metabolism: A transient signaling connection?. Plant Signal. Behav..

[49] Yang X., Srivastava R., Howell S.H., Bassham D.C. (2016). Activation of autophagy by unfolded proteins during endoplasmic reticulum stress. Plant J..

[50] Takatsuka C., Inoue Y., Higuchi T., Hillmer S., Robinson D.G., Moriyasu Y. (2011). Autophagy in tobacco BY-2 cells cultured under sucrose starvation conditions: Isolation of the autolysosome and its characterization. Plant Cell Physiol..

[51] Vanhee C., Zapotoczny G., Masquelier D., Ghislain M., Batoko H. (2011). The *Arabidopsis* multistress regulator TSPO is a heme binding membrane protein and a potential scavenger of porphyrins via an autophagy-dependent degradation mechanism. Plant Cell.

[52] Hachez C., Veljanovski V., Reinhardt H., Guillaumot D., Vanhee C., Chaumon F., Batoko H. (2014). The *Arabidopsis* abiotic stress-induced TSPO-related protein reduces cell-surface expression of the aquaporin PIP2; 7 through protein-protein interactions and autophagic degradation. Plant Cell.

[53] Williams B., Njaci I., Moghaddam L., Long H., Dickman M.B., Zhang X., Mundree S. (2015). Trehalose Accumulation Triggers Autophagy during Plant Desiccation. PLoS Genet..

[54] Yang Y.P., Hu L.F., Zheng H.F., Mao C.J., Hu W.D., Xiong K.P., Wang F., Liu C.F. (2013). Application and interpretation of current autophagy inhibitors and activators. Acta Pharmacol. Sin..

[55] Reumann S., Voitsekhovskaja O., Lillo C. (2010). From signal transduction to autophagy of plant cell organelles: Lessons from yeast and mammals and plant-specific features. Protoplasma.

[56] Uslu V.V., Grossmann G. (2016). The biosensor toolbox for plant developmental biology. Curr. Opin. Plant Biol..

[57] Cohen-Kaplan V., Livneh I., Avni1 N., Cohen-Rosenzweig C., Ciechanover A. (2016). The ubiquitin-proteasome system and autophagy: Coordinated and independent activities. Int. J. Biochem. Cell Biol..

